# Socioeconomic, Educational, Cultural, and Oral Health Practices Among Caregivers Declining Their Children’s Participation in School-Based Oral Health Promotion Programs: A Cross-Sectional Study

**DOI:** 10.3390/healthcare14101347

**Published:** 2026-05-14

**Authors:** Guilherme Assumpção Silva, Diego Augusto Amorim Cantão, Vitor Hugo Gonçalves Sampaio, Lourenço Vieira Tereza Canevari, Alessandra Marcondes Aranega, Wilson Galhego Garcia, Cristina Antoniali Silva, Daniela Atili Brandini

**Affiliations:** 1 Department of Diagnosis and Surgery, School of Dentistry, São Paulo State University (UNESP), Araçatuba 16015-050, SP, Brazil; guilherme.assumpcao@unesp.br (G.A.S.); diego.cantao@unesp.br (D.A.A.C.); vitor.sampaio@unesp.br (V.H.G.S.); locanevari@hotmail.com (L.V.T.C.); alessandra.aranega@unesp.br (A.M.A.); 2 Department of Basic Sciences, School of Dentistry, São Paulo State University (UNESP), Araçatuba 16015-050, SP, Brazil; wilson.galhego-garcia@unesp.br (W.G.G.); cristina.antoniali@unesp.br (C.A.S.)

**Keywords:** primary health care, community service-learning, health literacy, school dentistry, primary school

## Abstract

**Background/Objectives:** Oral health promotion in early childhood depends strongly on caregivers’ preventive care at home and educational centers. The aim of this study was to investigate socioeconomic, educational, cultural, and oral health factors associated with caregivers’ decisions to decline their children’s participation in school-based oral health promotion programs. **Methods:** Caregivers who did not authorize their children’s participation in the project were identified through school records and contacted using available information (name, telephone number, and email address). Participants were selected through simple random sampling. **Results:** Among the 58 caregivers included in the study, the main reasons reported were failure to return the consent form or missing the deadline (36.2%), considering the child too young (19.0%), already receiving private dental care (13.8%), not understanding the consent form (13.8%), not having received the document (10.3%), lack of trust in the professional (3.4%), and other reasons (3.4%). Higher income was significantly associated with higher educational level (*p* = 0.002), increased toothbrushing frequency (*p* = 0.007), shorter time since the last dental visit (*p* < 0.001), and lower levels of embarrassment related to oral health (*p* < 0.001). Additionally, lower-income caregivers were more likely to seek dental care only in the presence of problems (*p* = 0.046), while higher-income families were more likely to report private dental care as a reason for non-authorization (*p* < 0.001). **Conclusions:** These findings associate socioeconomic and educational inequalities with adverse effects on family oral health among parents, by reducing the frequency of preventive dental examinations and daily oral hygiene practices; and among children, by limiting authorization to participate in school-based oral health promotion programs. This evidence underscores that successful promotion of children’s oral health in educational settings requires addressing social disparities while strengthening caregivers’ knowledge and motivation to support participation.

## 1. Introduction

In early childhood, children’s health is profoundly dependent on the capacity of parents and caregivers to provide appropriate care. The knowledge, availability, beliefs, and resources of these individuals can significantly shape health outcomes, often determining whether preventive measures and medical guidance are effectively implemented, even when institutional support is available [1]. This dependency underscores the critical interplay between family practices and state interventions in safeguarding child well-being.

In many countries, child healthcare has been institutionalized as a structured practice designed to support families in monitoring children’s growth and development. Oral health promotion in early childhood education centers represents an important strategy for the prevention of dental diseases [2].

The environment of educational centers is regarded as a safe and appropriate setting for the implementation of oral health promotion, as it serves as a point of convergence among parents, educators, and healthcare professionals [3]. Within the school context, children receive education aimed at fostering autonomy in daily self-care practices through a structured routine. From this perspective, the “Happy Smile” extension project, implemented in the municipality of Araçatuba, São Paulo—Brazil, through the School of Dentistry of Araçatuba (FOA-UNESP), represents a public health initiative aimed at promoting equitable access to oral health services through educational activities and the provision of dental care delivered by undergraduate and postgraduate students [4].

Over recent decades, several countries have implemented structured services that integrate health education activities with dental procedures aimed at caries prevention [5,6]. Evidence indicates positive outcomes associated with these programs, particularly in increasing knowledge and improving oral hygiene-related behaviors [7,8].

During childhood, maintaining good oral health plays a fundamental role not only in children’s immediate quality of life but also in their well-being throughout adulthood [9]. The presence of untreated dental caries is associated with negative impacts on specific daily activities, such as difficulty eating and sleeping, and may consequently affect psychological and physical development [10,11].

Given the relevance of this topic, it is important to emphasize children’s dependence on the care provided within their family and social environments [12]. This dependence encompasses fundamental aspects such as adequate nutrition, hygiene practices, and access to medical and dental services. Due to developmental limitations, children lack autonomy in decisions related to their well-being, rendering them particularly vulnerable to the choices made by responsible adults.

Within this context, parents and caregivers play a central role in the establishment of healthy habits, directly influencing their children’s oral health [13,14]. The literature emphasizes that preventive education and dental care during childhood require consistent awareness-raising efforts and ongoing monitoring to ensure a healthy childhood and to promote comprehensive development [15].

Socioeconomic, cultural, and geographic factors may influence the ability of parents and caregivers to provide adequate care. Therefore, it is essential to recognize and address this vulnerability by ensuring equitable access to oral health services, education, and support for parents, caregivers and educators, as well as by implementing public policies that promote an environment conducive to children’s oral health and healthy development [16].

Despite the recognized importance of oral health promotion in early childhood, several barriers may influence parents and/or caregivers not to authorize their children’s participation in such programs. The aim of this study was to investigate socioeconomic, educational, cultural, and oral health factors associated with caregivers’ decisions to decline their children’s participation in school-based oral health promotion programs.

Addressing this research question will contribute to the development of more effective awareness and engagement strategies, thereby enhancing the impact of school-based interventions.

## 2. Materials and Methods

### 2.1. Ethical Aspects

The study was approved by the Research Ethics Committee (REC) under protocol number 79710124.7.0000.5420. All participants were informed about the objectives and procedures of the study, as well as about data confidentiality and their right to refuse or withdraw from participation at any time without penalty. Participation was granted upon signing the Informed Consent Form (ICF) (Appendix A).

The benefits arising from this research are not limited directly to the participants but are projected in the long term for the population. The study may identify relevant data regarding the needs, concerns, and challenges faced by parents or guardians of children with poor oral health, contributing to the prioritization of efforts and resources toward the most critical areas. Based on these findings, it will be possible to develop targeted intervention strategies aimed at overcoming barriers and promoting improvements in the oral health of children and their caregivers.

Another expected impact relates to public health, as the promotion of oral health from early childhood may reduce the incidence of dental diseases and contribute to improved quality of life in the future. Furthermore, by involving parents or guardians in the investigative process, the study fosters awareness and empowerment, enhancing understanding of the importance of pediatric oral care and strengthening informed decision-making regarding their children’s oral health.

### 2.2. Study Design, Sampling, and Recruitment

This is a cross-sectional study involving parents or caregivers of children in early childhood (0 to 5 years and 11 months of age) enrolled in childhood education centers participating in the “Happy Smile” project. These centers included nursery classes for infants, toddler groups (1–3 years), Preschool Stage 1 classes for children aged approximately 3–4 years, and Preschool Stage 2 classes for children aged approximately 5–6 years.

**Figure 1:**
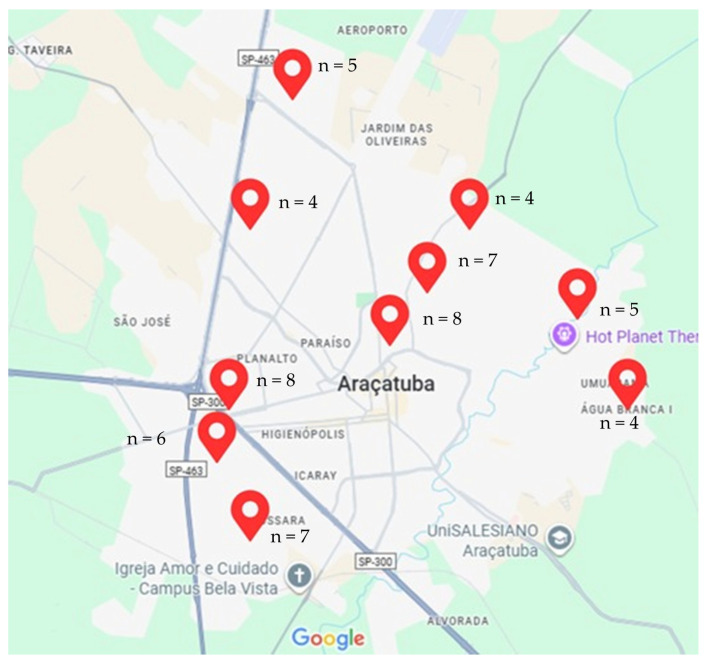
Geographic distribution of the educational centers included in the study across different regions of the municipality of Araçatuba, São Paulo, Brazil, showing the number of participants interviewed at each center.

Nineteen managers from early childhood education centers in the municipality of Araçatuba, São Paulo, who participated in the “Happy Smile” project in 2024 were invited to involve their respective communities in this research, as they served as the primary source of data and caregiver contact information. Of these, ten agreed to participate. The selected centers were located across different geographic regions of Araçatuba, including the north, south, east, west, and central areas of the municipality (Figure 1). This distribution was intended to ensure broader territorial representation and to include families from diverse socioeconomic and cultural backgrounds.

**Figure 2:**
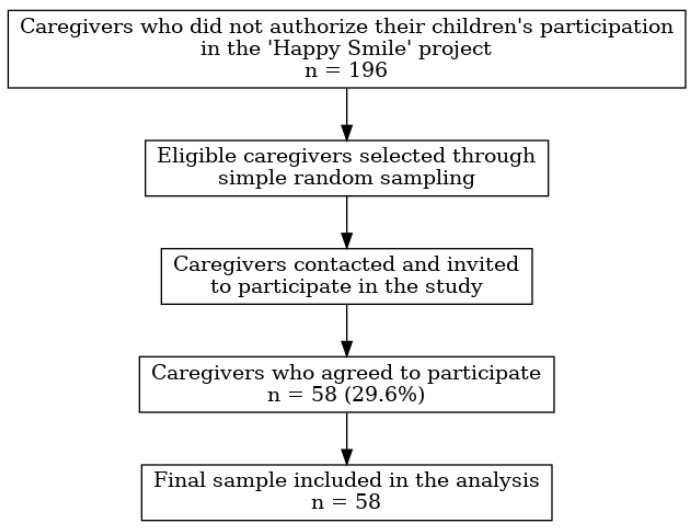
Flowchart of participant recruitment and inclusion in the study.

The sample size was estimated considering a finite population of 196 caregivers who did not authorize their children’s participation in the “Happy Smile” project. The calculation was performed using OpenEpi, version 3.01, an open-source calculator (Atlanta, GA, USA), adopting a confidence level of 90% (α = 0.10) and a margin of error of 10%. Since the expected prevalence of the outcome was unknown, a conservative proportion of 50% was used to maximize the sample size. Based on these parameters, the minimum required sample size was estimated to be 36 participants. The process of participant identification, recruitment, and inclusion in the study is illustrated in Figure 2. To minimize potential losses due to refusal or inability to contact participants, additional caregivers were approached during recruitment and invited to participate until the target sample size was achieved.

Initially, caregivers of children who did not participate in the “Happy Smile” project due to lack of authorization were identified. Subsequently, the education centers provided contact information of these caregivers (name, telephone number, and email address). Participants were selected through simple random sampling and contacted to be invited to participate, sign the Informed Consent Form (ICF), and complete the questionnaire (Appendix B). In cases of refusal, participants were replaced until the predetermined sample size was reached.

Caregivers who did not authorize their children’s participation in the dental care component of the project and who agreed to participate in the study were included. Those who could not be contacted or who declined to participate were excluded.

### 2.3. Data Collection and Instrument

Data collection was conducted by one of the authors (DAAC) through the administration of a structured questionnaire, either in person at the education centers where the children were enrolled or remotely, using real-time communication applications (WhatsApp or Google Hangouts).

The variables analyzed in the study encompassed four domains: socioeconomic characteristics, oral hygiene habits, oral healthcare practices, and psychosocial/cultural factors. Socioeconomic variables included family per capita income, categorized according to the number of Brazilian minimum wages earned per household member (per capita income): up to one minimum wage, up to two minimum wages, up to three minimum wages, and more than three minimum wages. This classification was used as a socioeconomic indicator, with lower income categories representing greater socioeconomic vulnerability and higher categories representing better socioeconomic conditions and caregiver educational level (incomplete secondary education, completed secondary education, incomplete higher education, and completed higher education). Variables related to oral hygiene habits included the oral hygiene tools used (toothbrush and toothpaste only, or toothbrush, toothpaste, and dental floss) and frequency of oral hygiene (none, once, twice, three times, or four or more times per day). Variables related to oral healthcare practices included time since the last dental visit (three months or less, more than three months, more than one year but less than two years, more than two years but less than five years, more than five years, or never had one) and reason for the last dental visit (routine check-up, pain or oral problem, other reason, or never had one). Psychosocial and cultural variables included embarrassment related to oral health (many times, frequently, occasionally, or never), trust in the professional, understanding of the informed consent form, perception that the child was too young to receive care, and whether the child was already receiving private dental care.

The primary outcome variable was the reason for non-authorization of the child’s participation in the “Happy Smile” project. These reasons included not receiving the informed consent form, not having time to return the form, not understanding the form, lack of trust in the professional, considering the child too young, already receiving private dental care, and other reasons.

These variables were obtained through a structured questionnaire specifically developed and administered to caregivers of children who were not authorized to participate in the ‘Happy Smile’ project.

Responses were recorded into predefined categories and subsequently coded for statistical analysis.

### 2.4. Statistical Analysis

Statistical analysis was performed using SPSS software version 21.0 (SPSS Inc., Chicago, IL, USA), adopting a significant level of 5% (α = 0.05). The data was organized according to the questionnaire responses (Appendix B). Associations between categorical variables were assessed using the chi-square test with Bonferroni post hoc adjustment, while correlations between categorical variables were evaluated using Spearman’s correlation test. Quantitative and ordinal variables obtained from the questionnaire were analyzed according to the predefined response categories. Variables such as family income, frequency of oral hygiene, time since the last dental visit, and embarrassment related to oral health were treated as categorical or ordinal variables in the statistical analyses. These groupings followed the response options of the questionnaire to facilitate interpretation and comparison between groups. Multivariate analysis was conducted using the Classification Tree technique to predict hierarchical associations among the data.

## 3. Results

**Figure 3:**
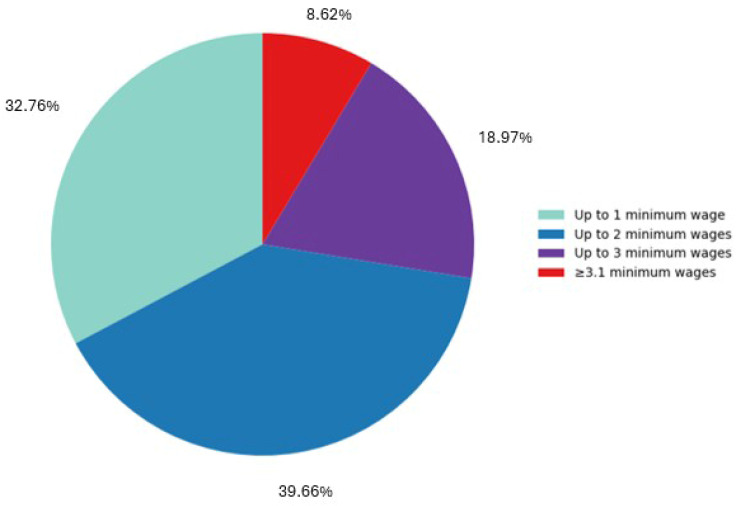
Distribution of family per capita income categories among the study participants.

A total of 58 caregivers of children whose participation in the “Happy Smile” project was not authorized were included in the study. The most prevalent income category among participants was up to two minimum wages (39.66%) (Figure 3). A significant correlation was identified between income and educational level (*p* = 0.002), indicating that families with higher income had higher levels of education (Table 1).

In relation to oral hygiene habits, 81.0% reported performing oral hygiene using only a toothbrush and toothpaste, while 19.0% also reported using dental floss. No significant association was found between income and the oral hygiene tools used (*p* = 0.649). Concerning tooth-brushing frequency, 37.9% reported brushing once a day, 37.9% twice a day, 17.2% three times a day, 3.4% four or more per day, and 3.4% did not brush daily. A significant correlation was observed between increased toothbrushing frequency and higher income (*p* = 0.007) (Table 1).

**Figure 4:**
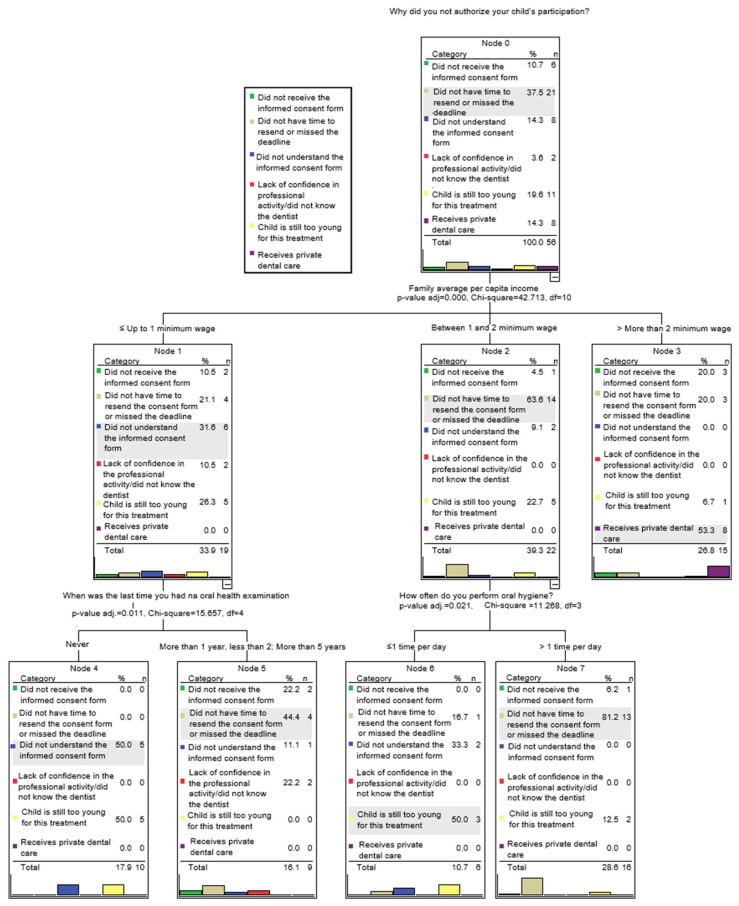
Characterization of the final tree showing the hierarchical associations between family income and reasons for non-authorization of children’s participation in oral health promotion activities at school. The CART algorithm was used for classification, with Gini impurity criteria and automatic pruning for a complexity cost of 0.01.

Regarding oral healthcare practices, only families earning three minimum wages or more had attended a preventive dental appointment within the past three months, whereas some families earning up to two minimum wages had never undergone a dental examination (Table 2). A significant Pearson correlation was observed between income and time since the last dental examination, indicating that higher-income caregivers were more likely to have had more recent dental visits (*p* < 0.001). Most participants sought dental care only when problems were present, particularly among caregivers with an income of one to two minimum wages (*p* = 0.046) (Table 2). Figure 4 illustrates the hierarchical associations among the variables related to caregivers’ decisions regarding participation in oral health promotion activities in early childhood education centers.

About psychosocial and cultural aspects, participants within the one minimum wage income category were the only group in which all individuals reported having felt embarrassed at some point about their oral health in the past year, with a substantial proportion reporting frequent feelings of embarrassment (Table 2). Pearson’s correlation analysis demonstrated that higher income was associated with lower levels of embarrassment related to oral health status (*p* < 0.001).

The main reasons reported for not authorizing the child’s participation in the project were failure to return the consent form or missing the deadline (36.2%), considering the child too young for care (19.0%), not understanding the consent form (13.8%), already receiving private dental care (13.8%), not having received the document (10.3%), lack of trust in the professional (3.4%), and other reasons (3.4%). Families earning three minimum wages or more were the only ones to report private dental care as the reason for non-authorization (*p* < 0.001) (Table 3).

## 4. Discussion

This study highlights the importance of family characteristics in shaping children’s dental care during early childhood. However, several limitations should be acknowledged. First, the sample was restricted to a single municipality, which may limit the generalizability of the findings to other regions with different socioeconomic, cultural, and educational contexts. Second, participation was voluntary, which may have introduced selection bias, as caregivers who agreed to participate could differ systematically from those who declined or were not reached. Third, the reliance on self-reported information obtained through questionnaires may have led to recall bias or social desirability bias, potentially influencing the accuracy of responses. Future research should expand the sample to include diverse regions, incorporate direct clinical assessments of children, and explore caregivers’ perceptions through qualitative approaches. Such strategies would strengthen the generalizability of findings and contribute to a broader understanding of cultural beliefs and health education practices.

Despite these limitations, this study contributes to the literature by specifically addressing caregivers who do not authorize their children’s participation in school-based oral health programs, a group that remains underexplored. By focusing on non-authorization behavior in a real-world public health context, the present study provides relevant insights into the socioeconomic, educational, and cultural barriers that may compromise the reach and effectiveness of preventive strategies in early childhood.

The socioeconomic and cultural profile of caregivers was identified as an important factor to access children’s oral health. Families with higher purchasing power and educational levels tend to adopt preventive practices and place greater value on oral health [17,18] opting for private care [19] which may compromise continuous monitoring and the development of healthy habits in children, as well as result in a lack of essential health data needed to map needs, optimize public policies, and strengthen healthcare provision [20]. Conversely, individuals with lower income reported less frequent or no dental visits [21]. In this study, the predominance of curative demand among caregivers who do not regularly seek dental care and who have a limited perception of the importance of prevention is consistent with previous studies indicating that, despite improvements in access, preventive dentistry has not yet been fully incorporated into routine practice [22,23].

Another relevant finding was that lower-income caregivers reported higher levels of embarrassment regarding their own oral health. This factor is generally associated with lower health literacy, reduced self-esteem, and social exclusion, which may decrease the likelihood of seeking care and contribute to resistance toward children’s dental treatment [24]. This dynamic reinforces patterns of non-authorization and consolidates emotional barriers that hinder the implementation of preventive policies [24].

Regarding oral hygiene habits, although the majority reported using toothbrush and toothpaste, the use of dental floss was less frequent, and toothbrushing frequency was below the recommended level in part of the sample. These findings highlight the need for educational interventions that consider families’ cultural beliefs and practices, as the adoption of appropriate oral hygiene behaviors is closely linked to how caregivers understand and value oral health care [25].

Barriers to authorizing oral health programs in early childhood education centers included logistical factors, such as missing the deadline or not receiving the informed consent form, as well as cultural perceptions, including the belief that conditions affecting primary teeth are not relevant and that dental care should be sought only when the child presents severe symptoms [19]. This resistance was more frequent among families with lower income and educational levels, highlighting informational and cultural barriers [26,27]. Lack of trust in oral health professionals also emerged as an important factor, often fueled by previous negative experiences and by the perception that professionals do not fully understand children’s specific needs [28].

Overall, the findings confirm the persistence of social inequalities in oral health, with a direct impact on access, frequency, and type of care sought. The presence of statistically significant associations between income, educational level, time since the last dental visit, reason for the last visit [29,30], and embarrassment related to oral health reinforces the need for public policies specifically targeted at more vulnerable populations. Such policies should include health education initiatives, expanded access to information, and awareness strategies that effectively engage families and consider their history of dental service utilization, as well as their experience with dental caries, in order to better understand and improve patterns of oral health service use [27,31]. In this context, it is essential to implement public policies focused on children, who are at a crucial stage of development for the establishment of healthy habits [16,18,19,32].

To increase caregivers’ participation, it is essential that oral health programs adopt a personalized approach, addressing doubts and concerns, reinforcing the importance of prevention from early childhood, and promoting trustworthy communication. Understanding the integration between oral health and primary healthcare is necessary to recognize the importance of oral health for overall health, with the aim of ensuring timely referral of children to dental care [33]. In addition, it is necessary to demystify health promotion in the school setting and strengthen the credibility of professionals by demonstrating the positive impact of participation in such programs on children’s well-being and the long-term development of healthy habits. However, such initiatives often do not receive adequate attention, particularly when considering the cost-effective allocation of limited healthcare resources—a reality that is especially relevant in developing countries. Therefore, this social analysis is important to support evidence-based decision-making in public policy [34].

During the data collection process, resistance was observed from some education centers in providing guardians’ contact information, citing bureaucratic issues or concerns regarding students’ exposure. This administrative barrier delayed certain stages of the research and highlights the need to align school administrators and staff regarding the benefits of community participation. Strengthening institutional partnerships, supported by clear protocols for the secure sharing of information, may represent a viable solution.

Parents or caregivers who do not authorize their children’s participation in oral health promotion activities in early childhood education centers generally belong to low-income households, do not attend preventive dental visits, report inadequate oral hygiene practices, and tend to undervalue oral care during early childhood. These findings reinforce the need for public policies that consider factors beyond the economic dimension, as parents take into account not only the financial aspects of pediatric treatments but also non-financial factors when making decisions [35]. From parents’ perspectives, the choice of pediatric dental treatment may be influenced by factors such as race, socioeconomic status, and educational level. In these cases, the use of dental services is often driven by the perception that the child’s condition is urgent [36].

Finally, the analysis reaffirms the relevance of early childhood health programs as instruments for reducing inequalities and should be accompanied by ongoing educational strategies tailored to the socioeconomic and cultural realities of the population served [37].

## 5. Conclusions

These findings associate socioeconomic and educational inequalities with adverse effects on family oral health among parents, by reducing the frequency of preventive dental examinations and daily oral hygiene practices; and among children, by limiting authorization to participate in school-based oral health promotion programs. This evidence underscores that successful promotion of children’s oral health in educational settings requires addressing social disparities while strengthening caregivers’ knowledge and motivation to support participation.

## Figures and Tables

**Table 1:** Association between income and education level, oral hygiene frequency and oral hygiene tools used. Chi-square test with Bonferroni post hoc adjustment and Spearman’s correlation test, α = 0.05.

	≤1 M.W.	≤2 M.W.	≤3 M.W.	≥3.1 M.W.	Total	Spearman’s Correlation *p* Value
Educational level						
Incomplete secondary education	3 a	4 a	0 a	0 a	7	
Completed secondary education	16 a	16 a	9 a	2 a	43	
Incomplete higher education	0 a	1 a	0 a	1 a	2	
Completed higher education	0 a	2 a, b	2 a, b	2 b	6	0.002
Frequency of oral hygiene						
None per day	2 a	0 a	0 a	0 a	2	
Once per day	12 a	6 a	4 a	0 a	22	
Twice per day	5 a	13 a	2 a	2 a	22	
Three times per day	0 a	4 a, b	4 b	2 b	10	
Four or more times per day	0 a	0 a	1 a	1 a	2	0.007
Oral hygiene tools used						
Toothbrush and toothpaste	17 a	17 a	9 a	4 a	47	
Toothbrush, toothpaste, and dental floss	2 a	6 a	2 a	1 a	11	0.649

Different letters indicate statistically significant differences between income categories after Bonferroni post hoc adjustment, whereas identical letters indicate no significant difference between groups.

**Table 2:** Income, dental visit patterns, and oral health embarrassment. Chi-square test with Bonferroni post hoc adjustment and Spearman’s correlation test, α = 0.05.

	≤1 M.W.	≤2 M.W.	≤3 M.W.	≥3.1 M.W.	Total	Spearman’s Correlation *p* Value
Time since the last dental visit						
Three months or less	0 a	0 a	0 a, b	2 b	2	
More than three months	0 a	4 a, b	4 b	1 a, b	9	
More than one year, but less than two years	6 a	5 a	5 a	2 a	18	
More than two years, but less than five years	0 a	2 a	1 a	0 a	3	
More than five years	3 a	7 a	1 a	0 a	11	
Never had one	10 a	5 a, b	0 b	0 a, b	15	0.001
Reason for the last dental visit						
For a routine check-up	0 a	2 a	3 a, b	3 b	8	
Something was wrong or causing pain	19 a	19 a, b	8 a, b	2 b	48	
Other	0 a	1 a	0 a	0 a	1	
Never had one	0 a	1 a	0 a	0 a	1	0.046
Embarrassment related to oral health						
Many times	9 a	0 b	0 b	0 a, b	9	
Frequently	2 a	4 a	2 a	0 a	8	
Occasionally	8 a, b	14 b	0 a	1 a, b	23	
Never	0 a	5 a, b	9 c	4 b, c	18	<0.001

Different letters indicate statistically significant differences between income categories after Bonferroni post-hoc adjustment, whereas identical letters indicate no significant difference between groups.

**Table 3:** Association between income and reasons for not authorizing child participation in the oral health promotion at Childhood Education Centers. Chi-square test with Bonferroni post hoc adjustment and Spearman’s correlation test, α = 0.05.

Reason for Non-Authorization	≤1 M.W.	≤2 M.W.	≤3 M.W.	≥3.1 M.W.	Total	*p* Value
Did not receive the informed consent form	2 a	1 a	3 a	0 a	6	
Did not have time to return the consent form	4 a	14 a	3 a	0 a	21	
Did not understand the consent form	6 a	2 a	0 a	0 a	8	
Lack of trust in the professional	2 a	0 a	0 a	0 a	2	
Considered the child too young	5 a	5 a	1 a	0 a	11	
Received private dental care	0 a	0 a	4 b	4 b	8	
Other	0 a	1 a	0 a	1 a	2	<0.001

Different letters indicate statistically significant differences between income categories after Bonferroni post-hoc adjustment, whereas identical letters indicate no significant difference between groups.

## Data Availability

The data presented in this study are available from the corresponding authors upon reasonable request. Requests must justify the need for the data, ensuring it is for research purposes, while adhering to privacy, legal, or ethical restrictions that prevented immediate, open access.

## Abbreviations

The following abbreviations are used in this manuscript:

REC	Research Ethics Committee
M.W	Minimum wage

## Appendix A

Research Title: Socioeconomic, Educational, and Cultural Factors Influencing Caregivers’ Authorization for Children’s Participation in School-Based Oral Health Promotion Activities
  Researcher’s Name:
  Supervisors’ Names:

1. Nature of the Research

You are being invited to participate in this research, which aims to understand the motivations and barriers related to maintaining your child’s oral health, in order to establish measures that may help promote it.

2. Research Participants

Parents and/or legal guardians of children included in the project “Projeto Sorriso Feliz: strengthening Primary Oral Health Care in very early and early childhood within Early Childhood Education in the State of São Paulo (DRS II).”

3. Participation in Research

By participating in this study, you will allow the researcher, Diego Augusto Amorim Cantão, to administer a questionnaire containing questions related to children’s oral health.

You are free to refuse participation and may withdraw from the study at any stage, without any penalty or harm to you or to the child.

You may request further information about the research at any time by contacting the project researcher at +55 (14) 99898-9024 or, if necessary, through the Research Ethics Committee.

4. About the Interviews

The interview will be conducted through a form prepared in accordance with the recommendations of the Research Ethics Committee (REC). After analysis, the results will be transformed into data aimed at generating benefits for the population as a whole.

5. Risks and Discomfort

Participation in this research does not violate any legal or ethical standards and follows the ethical guidelines for Research Involving Human Subjects in accordance with Resolution No. 466/12. None of the procedures used pose any risk to your dignity.

6. Confidentiality

All information collected in this study will be treated as strictly confidential. Only the researcher and their supervisors (and/or research team) will have access to your identity, and we commit to maintaining confidentiality when publishing the results of this research. If one participant chooses to remain anonymous, all others will follow the same criterion.

7. Benefits

By participating in this research, you will not receive any direct benefit. However, the results generated from this study may, in the long term, benefit the population as a whole.

The research may identify valuable data regarding the needs, concerns, and challenges faced by parents or caregivers of children with poor oral health, helping to direct efforts and resources to the areas where they are most needed. The researcher commits to disseminating the results obtained, while respecting the confidentiality of the collected information, as stated above.

8. Payment

You will not incur any expenses for participating in this research, nor will you receive any payment for your participation.

## Appendix B

Questionnaire for Parents/Guardians Who Did Not Authorize Their Child’s Participation in the School Oral Health Project

1. What is the average per capita household income (total household income divided by the number of residents)?
  (A) Up to 1 minimum wage.
  (B) Up to 2 minimum wages.
  (C) Up to 3 minimum wages.
  (D) More than 3 minimum wages.
2. What is your level of education?
  (A) Incomplete elementary school.
  (B) Complete elementary school.
  (C) Incomplete high school.
  (D) Complete high school.
  (E) Incomplete higher education.
  (F) Complete higher education.
3. When was the last time you had an oral health examination?
  (A) 3 months or less.
  (B) More than 3 months ago.
  (C) More than 1 year ago, but less than 2 years.
  (D) More than 2 years ago, but less than 5 years.
  (E) More than 5 years ago.
  (F) I have never had one.
4. What was the main reason for your last visit to the dentist?
  (A) For a routine check-up.
  (B) It was required by my workplace.
  (C) Something was wrong or painful.
  (D) For follow-up care.
  (E) Other.
5. If you were unable to receive dental care during your last visit, what was the reason?
  (A) I could not afford it.
  (B) Insurance does not cover oral health care.
  (C) The dental health center is too far away.
  (D) I did not have time.
  (E) I was afraid to go.
6. During the past year, how often have you experienced discomfort in the following parts of your mouth?
  (A) Teeth
  (B) Gums
  (C) Tongue
  (D) Jaws
7. During the past year, how often have you felt embarrassed or ashamed because of oral health problems?
  (A) Many times.
  (B) Frequently.
  (C) Occasionally.
  (D) Never.
8. What do you use to clean your teeth?
  (A) Dental floss only.
  (B) Toothbrush and toothpaste.
  (C) Toothbrush, toothpaste, and dental floss.
  (D) Other.
9. How often do you perform oral hygiene?
  (A) Never.
  (B) Once a day.
  (C) Twice a day.
  (D) Three times a day.
  (E) Four times or more per day.
10. What was the reason you did not authorize your child’s participation in the “Sorriso Feliz” project at school?
  (A) I did not receive the informed consent form.
  (B) I did not have time to return the form/I missed the submission deadline.
  (C) I did not understand the informed consent form.
  (D) Lack of trust in the professional activity because I do not know the dentist.
  (E) My child is still too young for this type of treatment.
  (F) I checked my child’s mouth and saw there were no cavities.
  (G) My child does not complain of pain and is eating normally.
  (H) It is not a priority for my family.
  (I) Other: __________________________
